# Supplementary material for: Assembly of a Benthic Microbial Community in a Eutrophic Bay with a Long History of Oyster Culturing
Source: Microorganisms. 2021 Sep 24;9(10):2019. doi: 10.3390/microorganisms9102019 (PMC8536970; doi:10.3390/microorganisms9102019)
Supplement: Supplementary file 1 [file microorganisms-09-02019-s001.zip › microorganisms-1386570-supplementary.pdf]

Supplementary Materials

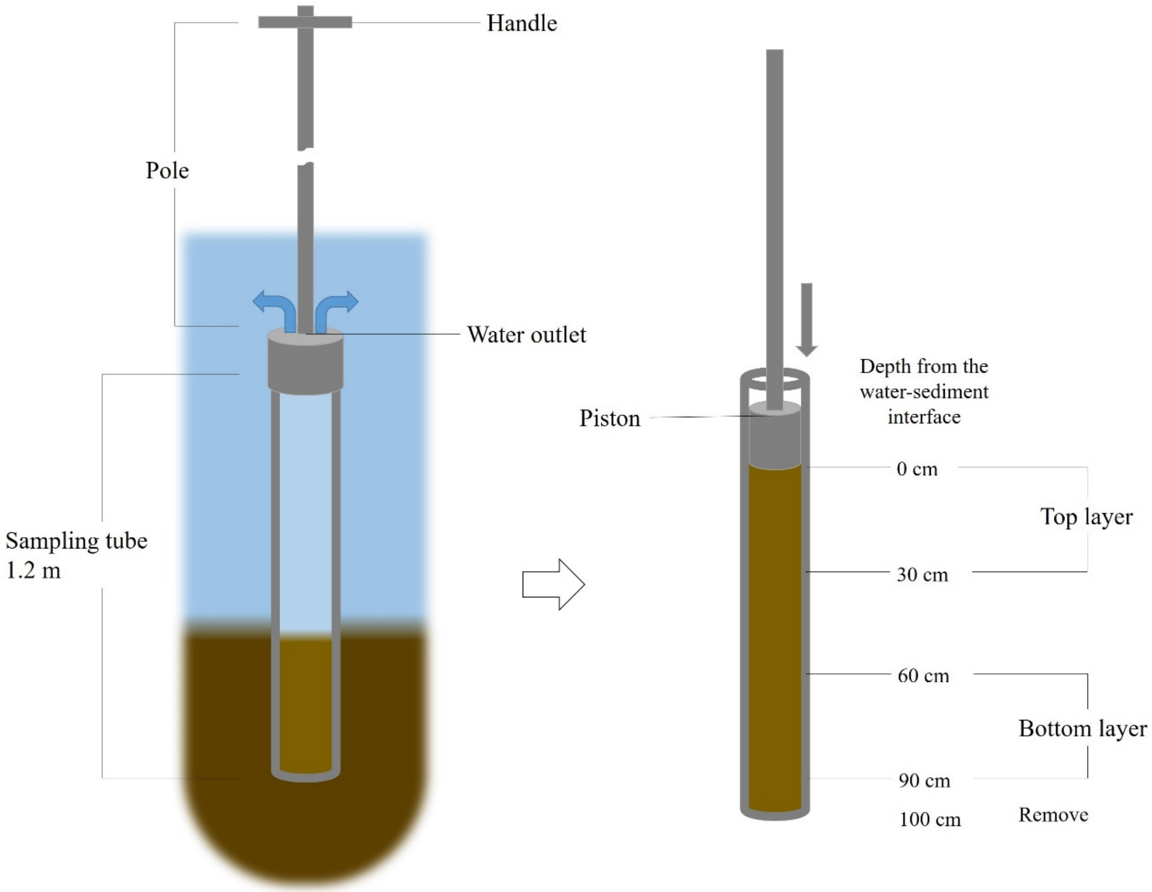

Figure S1. Schematic view of the sampling device (left) and the sediment core (right). Sediment sample showed in brown and water in light blue. A handle and a pole were used to push down the sampling tube into the sediment and lift it out. The bottom 10 cm or more of each core was removed as it expored in air too long. The rest of the sediment was devided for the top layer and the bottom layer samples as depicted.

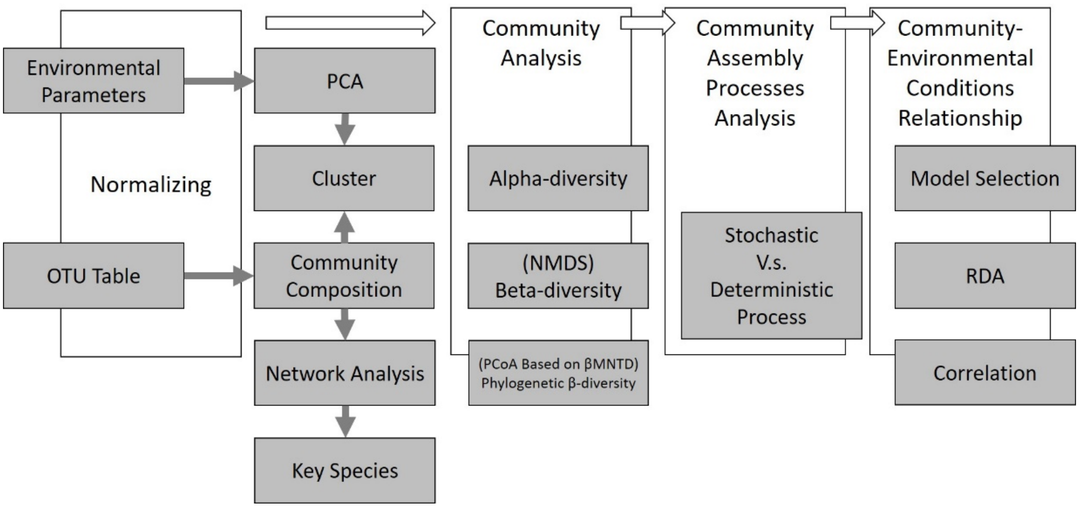

Figure S2. Work flow of main analysis methods.

Table S1. Environmental parameters.

Sample	pH	ORP	moisture	NH_4^+(mg*kg^{-1})-N	TN%	TOC%
TO1	7.463	-247.5	0.66	3.2935	0.2242	1.8543
TO2	7.663	-249.5	0.62	1.7845	0.1717	1.2695
TO3	7.520	-198.6	0.62	1.9715	0.2242	1.6879
TO4	7.423	-239.7	0.64	0.992	0.2290	1.6879
TO5	7.123	-269	0.58	0.76	0.2099	1.5976
TO6	7.397	-272.4	0.63	3.078	0.2385	1.7449
TO7	7.640	-270.8	0.62	2.8865	0.2337	1.8495
TO8	7.357	-280.4	0.59	0.929	0.1813	1.5262
BO1	7.983	-70.9	0.45	1.254	0.1336	1.4074
BO2	7.737	-113.6	0.42	0.6195	0.1288	0.9604
BO3	7.757	-70.1	0.43	0.492	0.1526	1.1221
BO4	7.813	-226.2	0.38	0.4085	0.1336	1.0318
BO5	7.333	-160.6	0.42	0.357	0.1240	0.8368
BO6	7.837	-53.8	0.42	0.719	0.1193	0.8558
BO7	7.727	-151.3	0.45	1.009	0.1288	1.0318
BO8	7.613	-140.7	0.41	0.2375	0.1002	0.7798
TN1	7.427	-131.6	0.55	0.5005	0.1431	0.9747
TN2	7.167	-107.2	0.49	0.3365	0.1526	1.3028
TN3	7.470	-155	0.41	0.2835	0.1622	1.4739
TN4	7.310	-243.9	0.52	0.514	0.2147	1.8020
TN5	7.717	-178	0.54	0.3385	0.1622	1.2267
TN6	7.497	-215.5	0.54	0.5605	0.1717	1.2695
TN7	7.480	-273.9	0.56	0.28	0.1670	1.2457
TN8	7.413	-166.5	0.50	0.4085	0.1383	1.2219
BN1	7.673	-140.5	0.40	0.623	0.0954	0.6704
BN2	7.667	-198.5	0.51	0.94	0.1336	0.9509
BN3	7.667	-232.4	0.52	0.4865	0.1622	1.1506
BN4	7.747	-234.1	0.51	0.85	0.1622	1.2077
BN5	7.910	-182.6	0.52	0.4985	0.1574	1.0175
BN6	7.560	-171.3	0.51	1.2815	0.1336	0.9034
BN7	7.497	-195	0.51	1.5075	0.1336	0.8273
BN8	7.600	-114.9	0.51	1.1605	0.1574	1.3028

Table S2. Correlation of environmental indexes of sediments across all groups. Red: positively related; blue: negatively related; star: significantly related. (n=32, *: p<0.1, **: p<0.05, ***: p<0.01)

	ORP	moisture	NH_4^+	TN	TOC
pH	0.388 *	-0.386 *	-0.034	-0.414 *	-0.389 *
ORP		**-0.687** ***	-0.382 *	**-0.666** ***	**-0.54** **
moisture			**0.647** ***	**0.835** ***	**0.71** ***
NH_4^+				**0.608** ***	**0.557** ***
TN					**0.931** ***

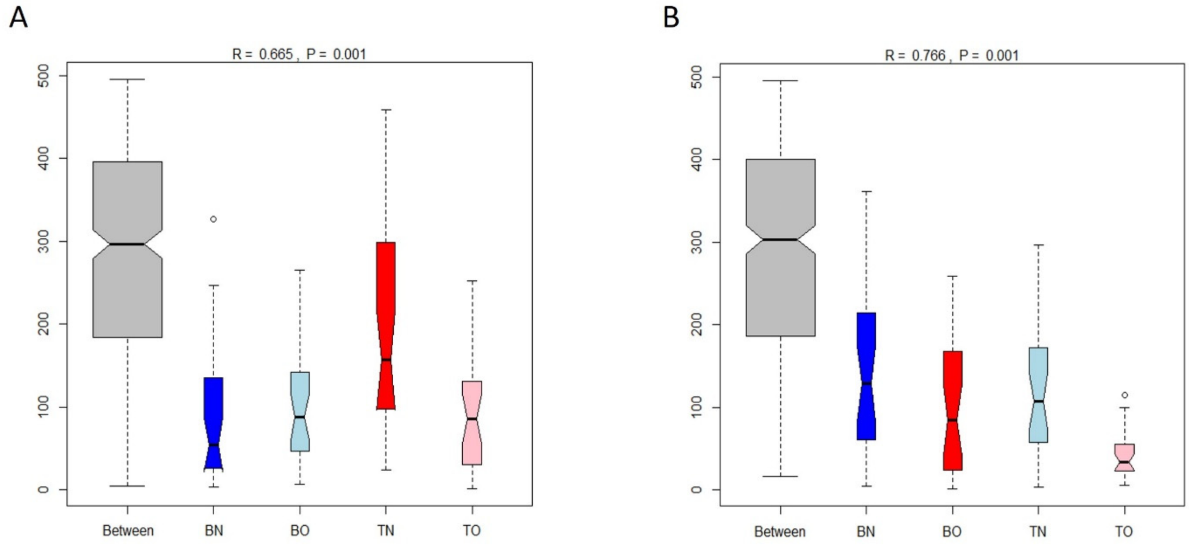

Figure S3. ANOSIM analysis of environmental parameters and species abundance of four groups of samples. (A) Environmental parameters; (B) species. Differences between, gray, and within groups (top layer of oyster-culture site, TO, pink, bottom layer of oyster-culture site, BO, red, top layer of non-culture site, TN, light blue, and bottom layer of non-culture site, BN, blue). The same colorings were adopted in figures below when refer to sample groups.(n=8, $p < 0.05$. Error bar, mean ± s.d.)

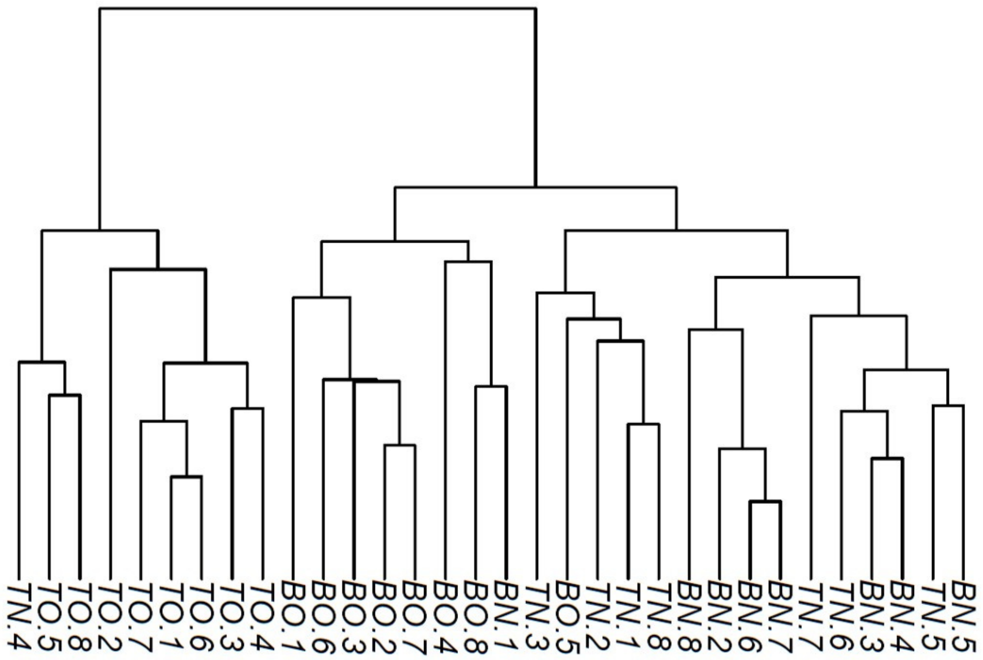

Figure S4. Cluster based on environmental parameters.

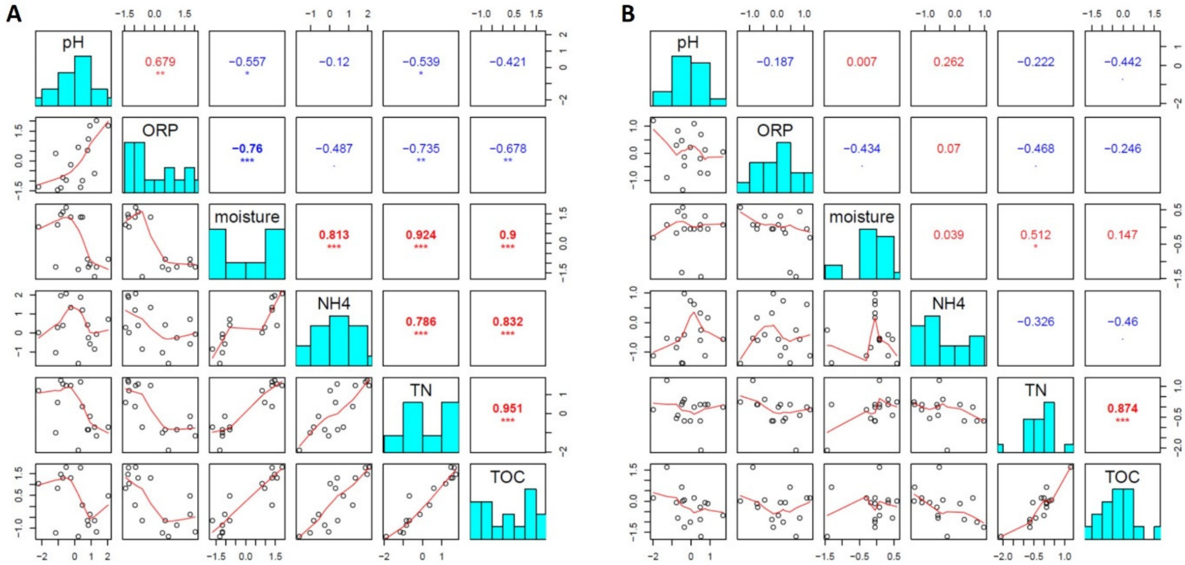

Figure S5. Correlation of environmental indexes of sediments. (A) Oyster culture area; (B) control area. Histograms on the dioganol: distribution of given parameters; scatter diagrams in the lower left rectangular triangle: relation of any pair of environmental parameters; upper right part: correlation coefficients, blue corlor indicating negative and red color positive correlation and stars significant corrlation. (n=16, *: p<0.1, **: p < 0.05, ***: p<0.01)

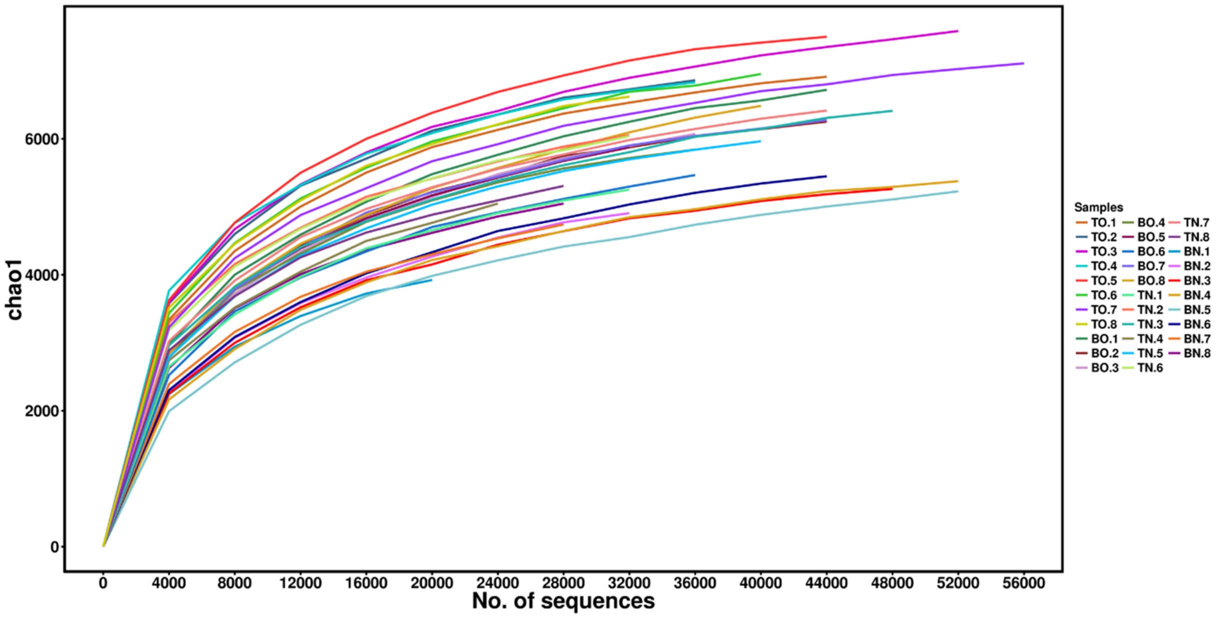

Figure S6. Rarefaction curve. The curves change more slowly as reads accumulate, showing that the sampling volume was reasonable.

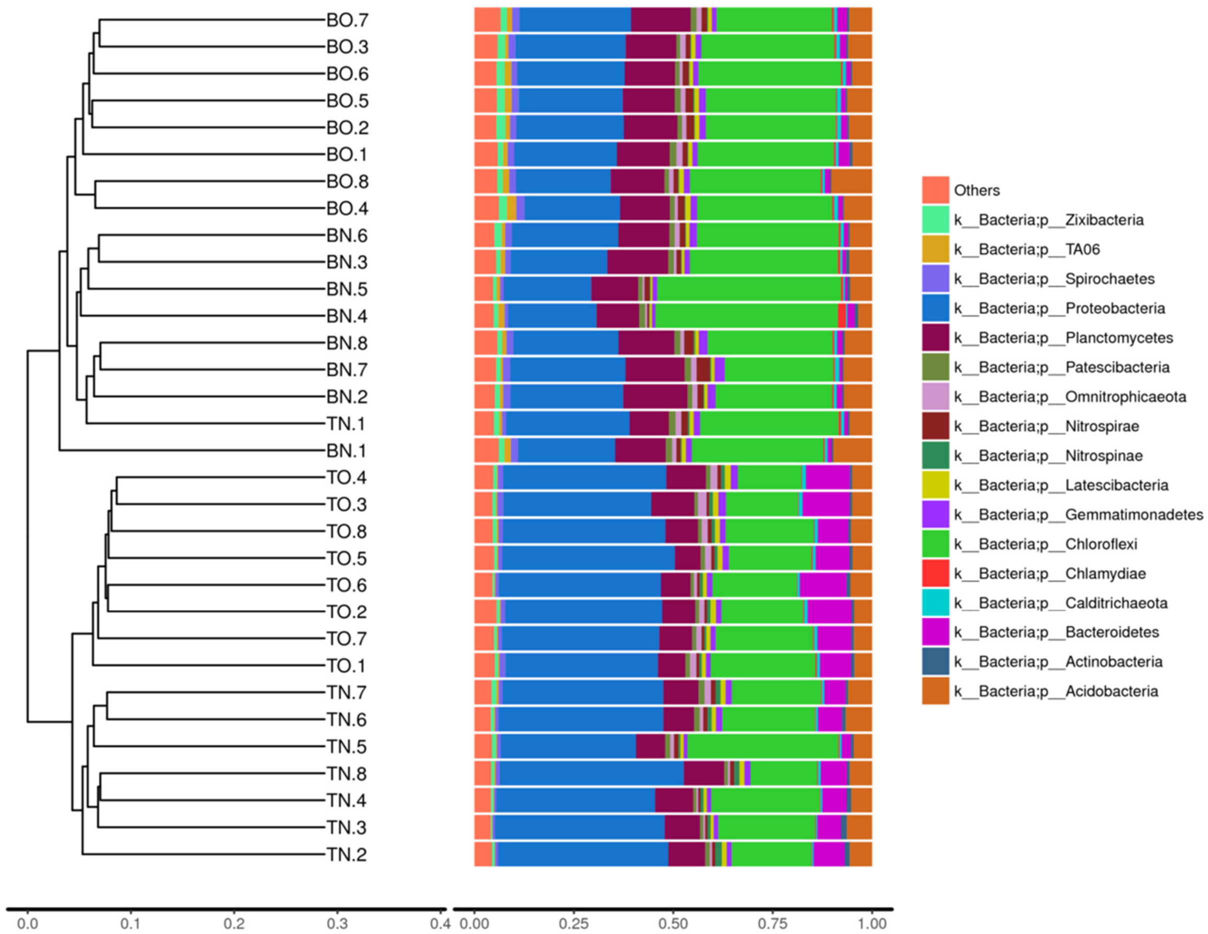

Figure S7. Relative abundance (right) and cluster (left) of microbial communities of samples.

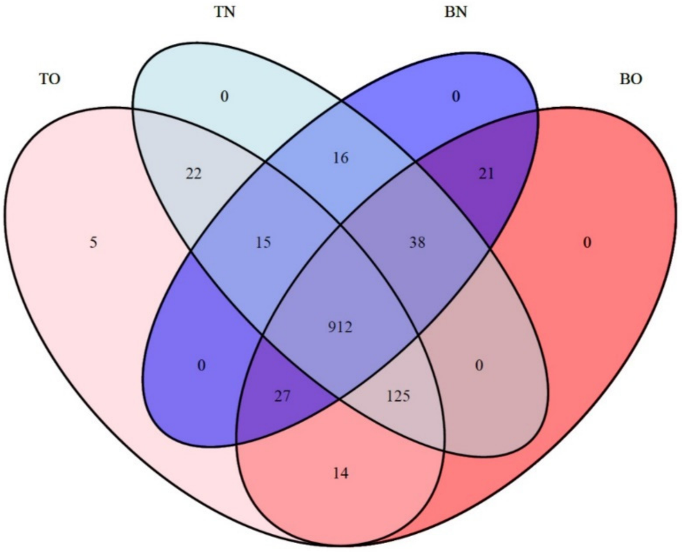

Figure S8. Venn diagram. A given species was "present" in a group when it was present in at least 4 out of 8 samples of the group. Numbers in overlaping areas were numbers of shared species, other wise the numbers stand for group specific species in according group.

Table S3. βNTI of the microbial community.

	BO.1	TO.2	BO.2	TO.3	BO.3	TO.4	BO.4	TO.5	BO.5	TO.6	BO.6	TO.7	BO.7	TO.8	BO.8	TN.1	BN.1	TN.2	BN.2	TN.3	BN.3	TN.4	BN.4	TN.5	BN.5	TN.6	BN.6	TN.7	BN.7	TN.8	BN.8
**TO.1**	1.71	-0.25	3.77	0.31	0.38	1.90	2.91	0.59	1.68	2.92	2.73	0.17	1.63	-0.18	2.77	2.61	2.74	1.30	1.52	1.89	2.80	2.36	3.40	2.17	2.49	1.74	2.50	1.64	1.43	2.41	2.54
**BO.1**		2.55	2.51	2.08	2.27	3.71	1.13	1.81	3.22	4.57	3.17	0.72	2.42	0.98	1.71	2.97	2.84	1.89	3.01	1.93	2.63	2.18	1.50	1.38	1.90	1.62	1.69	1.68	3.02	2.16	2.06
**TO.2**			3.59	0.75	2.26	1.62	2.18	1.46	2.27	3.47	2.69	1.60	3.30	1.80	0.77	2.86	2.21	1.36	2.68	1.25	3.40	1.48	2.84	1.40	2.57	1.39	3.13	1.60	2.43	1.72	2.60
**BO.2**				3.51	2.41	5.87	1.34	4.86	1.49	4.57	3.24	1.62	2.41	2.41	0.04	2.41	2.77	3.49	1.90	2.89	2.77	2.41	2.84	2.96	2.05	2.61	2.03	2.09	2.63	2.50	2.94
**TO.3**					1.91	1.52	0.83	1.67	1.59	3.33	2.84	0.18	4.02	-0.79	0.29	3.68	1.69	1.91	3.75	1.58	4.15	2.00	3.83	2.47	3.25	1.88	3.31	1.63	2.76	2.02	2.68
**BO.3**						4.14	-0.05	2.20	2.52	2.58	1.79	0.67	1.95	0.20	1.31	2.08	2.09	2.32	2.00	1.78	1.82	1.96	2.18	2.36	1.97	2.10	1.99	1.67	2.45	1.48	2.10
**TO.4**							2.20	2.23	3.07	3.01	3.53	2.12	6.29	3.86	1.37	3.93	1.98	1.41	3.58	1.18	4.65	1.52	3.99	1.74	3.31	1.67	4.24	1.60	3.58	1.83	3.22
**BO.4**								1.81	1.45	1.54	0.69	-0.01	0.33	1.73	1.49	1.35	1.71	1.94	1.41	1.62	1.11	1.52	1.73	2.05	1.06	1.88	0.95	1.38	1.51	0.97	1.36
**TO.5**									1.58	3.13	2.18	0.77	2.28	1.17	0.89	2.59	2.24	1.35	2.27	1.35	2.94	1.50	2.74	1.29	2.35	1.51	2.77	1.60	2.19	1.66	2.49
**BO.5**										1.31	1.23	-0.06	0.10	1.54	-0.04	2.29	2.40	2.63	1.86	2.20	2.17	2.06	2.54	2.63	2.22	2.27	1.59	1.62	1.94	1.39	1.71
**TO.6**											2.92	2.62	5.17	2.81	0.52	3.41	1.32	1.65	3.12	1.28	4.75	1.58	3.02	2.12	3.22	1.87	3.64	1.68	3.07	1.93	3.21
**BO.6**												1.02	1.98	1.40	1.08	1.97	2.52	2.97	1.96	1.97	1.94	1.78	3.04	2.26	1.91	2.41	1.80	2.04	1.77	1.59	2.06
**TO.7**													2.04	0.31	-0.19	2.60	2.26	2.48	1.59	2.20	2.61	2.09	1.75	2.83	1.69	2.67	2.08	2.20	1.74	2.94	1.62
**BO.7**														1.81	0.19	2.54	2.52	2.77	2.12	2.45	2.38	2.66	2.44	2.52	1.89	2.55	1.86	2.25	2.12	2.31	1.69
**TO.8**															1.66	2.04	2.43	1.67	1.90	1.46	2.16	1.59	1.16	2.00	1.70	1.67	2.05	1.36	1.55	1.67	2.03
**BO.8**																2.03	2.47	2.61	2.35	2.09	2.40	1.88	3.24	2.17	2.26	2.51	1.80	1.91	2.16	1.55	2.31
**TN.1**																	0.90	2.92	0.68	2.80	1.25	1.55	3.41	1.99	1.29	2.00	0.91	1.44	0.54	1.77	1.62
**BN.1**																		1.25	1.16	0.94	0.61	0.52	0.97	1.74	1.83	1.50	-0.33	1.20	1.25	1.01	0.97
**TN.2**																			2.80	0.88	3.98	1.48	0.96	1.50	2.23	0.75	2.09	1.41	1.59	1.21	2.64
**BN.2**																				1.50	-0.02	0.79	0.50	1.02	0.35	1.47	0.00	1.63	0.48	1.34	0.92
**TN.3**																					1.69	1.18	1.18	1.83	2.05	0.97	2.25	1.94	1.18	1.69	2.25
**BN.3**																						1.18	0.14	1.82	1.68	2.53	2.55	2.17	1.27	1.50	0.91
**TN.4**																							1.52	0.91	2.09	1.46	2.08	1.17	0.96	1.38	1.33
**BN.4**																								2.23	3.40	1.02	3.27	1.20	1.61	0.03	0.75
**TN.5**																									2.70	0.97	1.06	0.51	0.42	1.49	0.85
**BN.5**																										2.27	0.68	1.61	0.62	1.49	0.71
**TN.6**																											1.29	0.90	1.18	0.07	0.44
**BN.6**																												1.68	-0.05	2.59	0.66
**TN.7**																													1.62	0.74	1.29
**BN.7**																														1.18	0.65
**TN.8**																															0.98

Table S4. RC_{bray} of the microbial community.

	BO.1	TO.2	BO.2	TO.3	BO.3	TO.4	BO.4	TO.5	BO.5	TO.6	BO.6	TO.7	BO.7	TO.8	BO.8	TN.1	BN.1	TN.2	BN.2	TN.3	BN.3	TN.4	BN.4	TN.5	BN.5	TN.6	BN.6	TN.7	BN.7	TN.8	BN.8
**TO.1**	1.00	0.91	1.00	1.00	1.00	1.00	1.00	0.89	1.00	1.00	1.00	0.25	1.00	0.96	1.00	1.00	1.00	1.00	1.00	1.00	1.00	1.00	1.00	1.00	1.00	1.00	1.00	1.00	1.00	1.00	1.00
**BO.1**		1.00	1.00	1.00	1.00	1.00	1.00	1.00	1.00	1.00	1.00	1.00	0.98	1.00	1.00	1.00	1.00	1.00	1.00	1.00	1.00	1.00	1.00	1.00	1.00	1.00	1.00	1.00	1.00	1.00	1.00
**TO.2**			1.00	0.92	1.00	-0.10	1.00	-0.41	1.00	-0.34	1.00	-0.65	1.00	-0.37	1.00	1.00	1.00	1.00	1.00	1.00	1.00	1.00	1.00	1.00	1.00	1.00	1.00	1.00	1.00	1.00	1.00
**BO.2**				1.00	-0.97	1.00	1.00	1.00	0.64	1.00	-1.00	1.00	0.53	1.00	1.00	0.89	1.00	1.00	0.99	1.00	0.98	1.00	1.00	1.00	1.00	1.00	0.76	1.00	0.82	1.00	0.98
**TO.3**					1.00	-0.06	1.00	0.92	1.00	0.86	1.00	1.00	1.00	0.95	1.00	1.00	1.00	1.00	1.00	1.00	1.00	1.00	1.00	1.00	1.00	1.00	1.00	1.00	1.00	1.00	1.00
**BO.3**						1.00	1.00	1.00	0.99	1.00	0.14	1.00	-0.49	1.00	1.00	0.97	1.00	1.00	1.00	1.00	0.90	1.00	1.00	1.00	0.98	1.00	0.63	1.00	1.00	1.00	1.00
**TO.4**							1.00	-0.36	1.00	-0.15	1.00	0.89	1.00	0.70	1.00	1.00	1.00	1.00	1.00	1.00	1.00	1.00	1.00	1.00	1.00	1.00	1.00	1.00	1.00	1.00	1.00
**BO.4**								1.00	0.75	1.00	1.00	1.00	1.00	1.00	0.09	1.00	0.61	1.00	1.00	1.00	1.00	1.00	1.00	1.00	1.00	1.00	1.00	1.00	1.00	1.00	1.00
**TO.5**									1.00	-0.03	1.00	0.33	1.00	-0.74	1.00	1.00	1.00	1.00	1.00	1.00	1.00	1.00	1.00	1.00	1.00	1.00	1.00	1.00	1.00	1.00	1.00
**BO.5**										1.00	0.14	1.00	1.00	1.00	1.00	1.00	1.00	1.00	0.67	1.00	1.00	1.00	1.00	1.00	1.00	1.00	1.00	1.00	0.24	1.00	0.27
**TO.6**											1.00	0.87	1.00	0.72	1.00	1.00	1.00	1.00	1.00	1.00	1.00	1.00	1.00	1.00	1.00	1.00	1.00	1.00	1.00	1.00	1.00
**BO.6**												1.00	0.86	1.00	1.00	1.00	1.00	1.00	0.99	1.00	0.97	1.00	1.00	1.00	1.00	1.00	0.77	1.00	0.93	1.00	0.97
**TO.7**													1.00	-0.06	1.00	1.00	1.00	1.00	1.00	1.00	1.00	1.00	1.00	1.00	1.00	1.00	1.00	1.00	1.00	1.00	1.00
**BO.7**														1.00	1.00	1.00	1.00	1.00	1.00	1.00	0.97	1.00	1.00	1.00	1.00	1.00	0.98	1.00	1.00	1.00	1.00
**TO.8**															1.00	1.00	1.00	1.00	1.00	1.00	1.00	1.00	1.00	1.00	1.00	1.00	1.00	1.00	1.00	1.00	1.00
**BO.8**																1.00	-1.00	1.00	1.00	1.00	1.00	1.00	1.00	1.00	1.00	1.00	1.00	1.00	1.00	1.00	1.00
**TN.1**																	1.00	1.00	0.97	1.00	0.96	1.00	1.00	1.00	0.79	1.00	0.38	1.00	0.89	1.00	0.99
**BN.1**																		1.00	1.00	1.00	1.00	1.00	1.00	1.00	1.00	1.00	1.00	1.00	1.00	1.00	1.00
**TN.2**																			1.00	0.83	1.00	0.99	1.00	1.00	1.00	0.97	1.00	1.00	1.00	0.99	1.00
**BN.2**																				1.00	1.00	1.00	1.00	1.00	1.00	1.00	0.86	1.00	-0.99	1.00	-0.97
**TN.3**																					1.00	-1.00	1.00	1.00	1.00	0.84	1.00	1.00	1.00	0.35	1.00
**BN.3**																						1.00	0.95	1.00	-0.83	1.00	-0.71	1.00	0.99	1.00	0.99
**TN.4**																							1.00	1.00	1.00	0.99	1.00	1.00	1.00	0.73	1.00
**BN.4**																								1.00	0.96	1.00	1.00	1.00	1.00	1.00	1.00
**TN.5**																									1.00	1.00	1.00	1.00	1.00	1.00	1.00
**BN.5**																										1.00	-0.48	1.00	1.00	1.00	0.99
**TN.6**																											1.00	0.54	1.00	0.57	1.00
**BN.6**																												1.00	0.29	1.00	0.88
**TN.7**																													1.00	0.89	1.00
**BN.7**																														1.00	-1.00
**TN.8**																															1.00

Table S5. Network indexes of each group.

Numbers in parentheses indicate the correlation threshold.

**Network Indexes**	**TO(0.930)**	**BO(0.940)**	**TN(0.940)**	**BN(0.950)**
Total nodes	772	744	712	554
Total links	991	2203	2435	917
R square of power-law	0.947	0.889	0.886	0.907
Average degree (avgK)	2.567	5.922	6.84	3.31
Average clustering coefficient (avgCC)	0.149	0.24	0.224	0.189
Average path distance (GD)	10.192	5.45	5.2	6.674
Geodesic efficiency (E)	0.133	0.241	0.243	0.192
Harmonic geodesic distance (HD)	7.499	4.154	4.107	5.215
Maximal degree	26	49	66	23
Nodes with max degree	OTU430	OTU253	OTU212	OTU167
Centralization of degree (CD)	0.03	0.058	0.083	0.036
Maximal betweenness	28707.453	11588.358	12716.819	9945.075
Nodes with max betweenness	OTU430	OTU13220	OTU221	OTU687
Centralization of betweenness (CB)	0.092	0.04	0.047	0.061
Maximal stress centrality	681975	1388380	391519	186561
Nodes with max stress centrality	OTU757	OTU644	OTU221	OTU20
Centralization of stress centrality (CS)	2.219	4.878	1.478	1.176
Maximal eigenvector centrality	0.463	0.209	0.206	0.261
Nodes with max eigenvector centrality	OTU430	OTU900	OTU708	OTU588
Centralization of eigenvector centrality (CE)	0.456	0.197	0.192	0.246
Density (D)	0.003	0.008	0.01	0.006
Reciprocity	1	1	1	1
Transitivity (Trans)	0.233	0.42	0.313	0.277
Connectedness (Con)	0.383	0.369	0.514	0.428
Efficiency	0.994	0.981	0.984	0.99
Hierarchy	0	0	0	0
Lubness	1	1	1	1
Module	111	124	88	83
Modularity	0.879	0.508	0.495	0.752

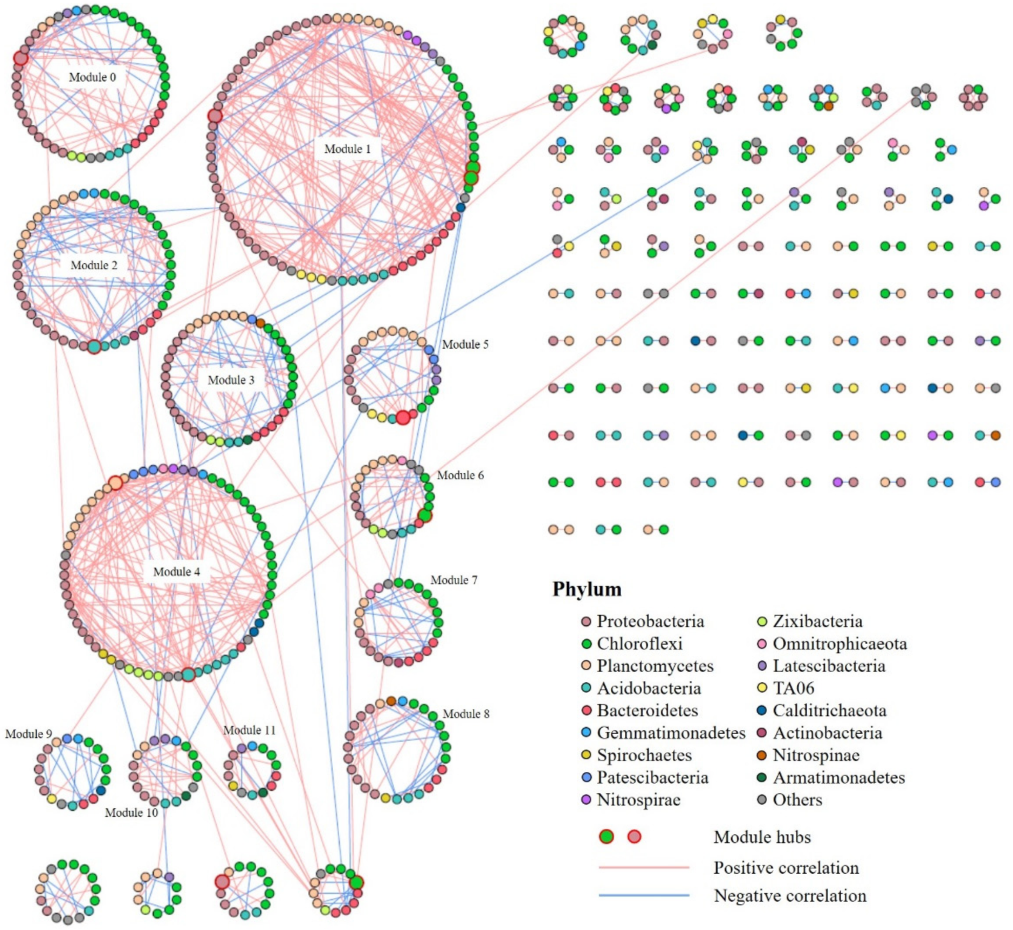

Figure S9. Network of microbial community of TO group.

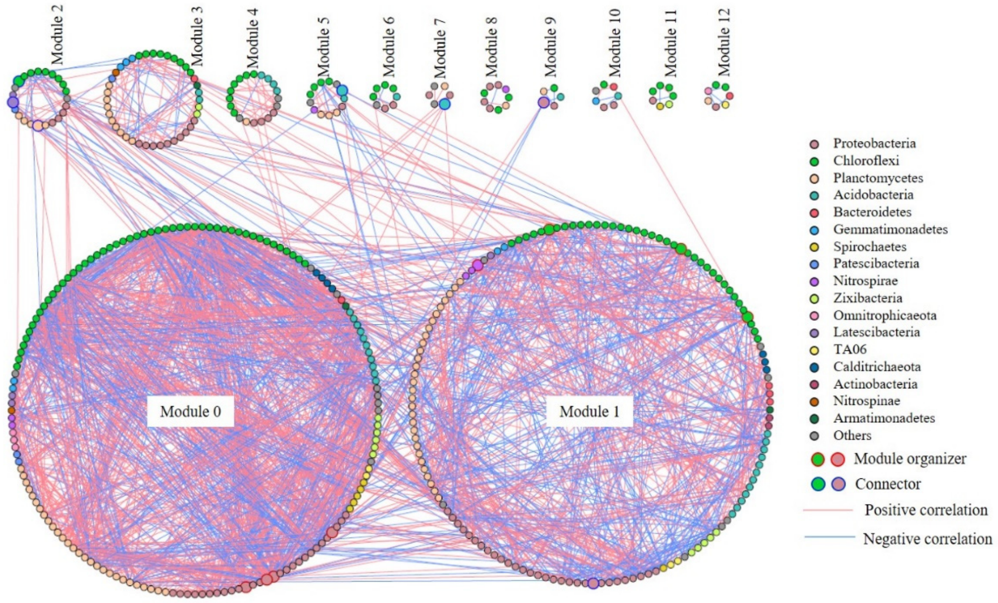

Figure S10. Network of microbial community of BO group.

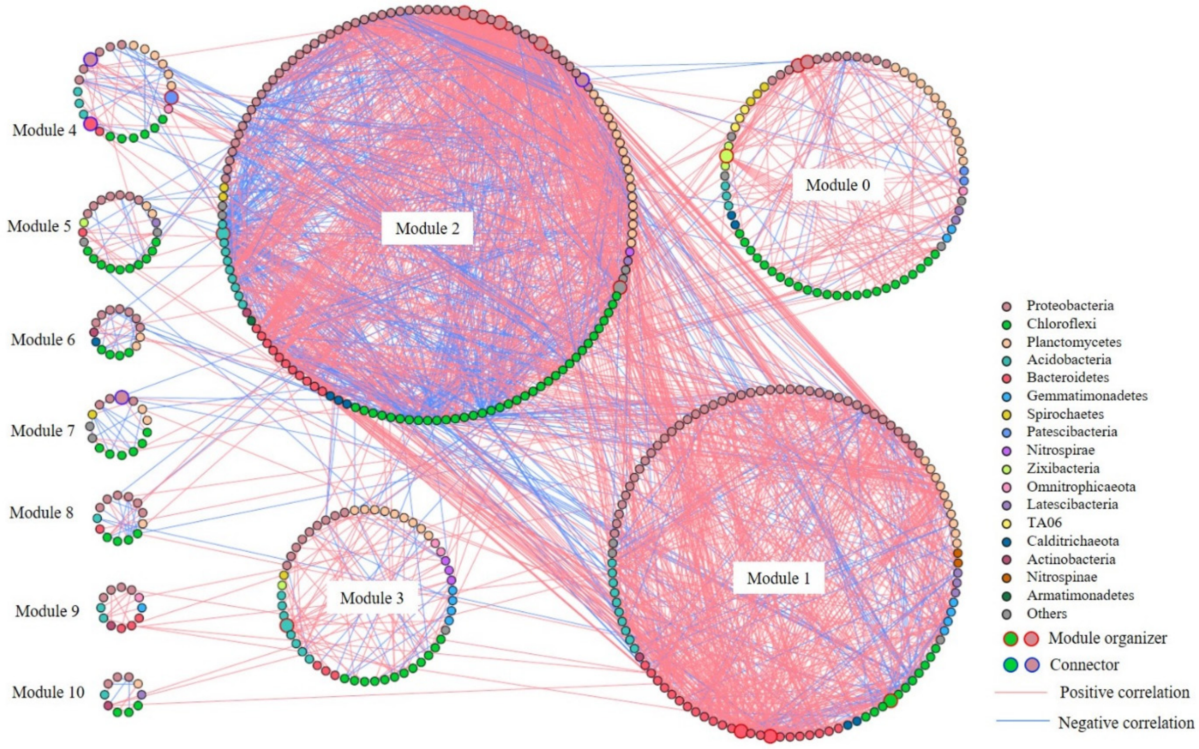

Figure S11. Network of microbial community of TN group.

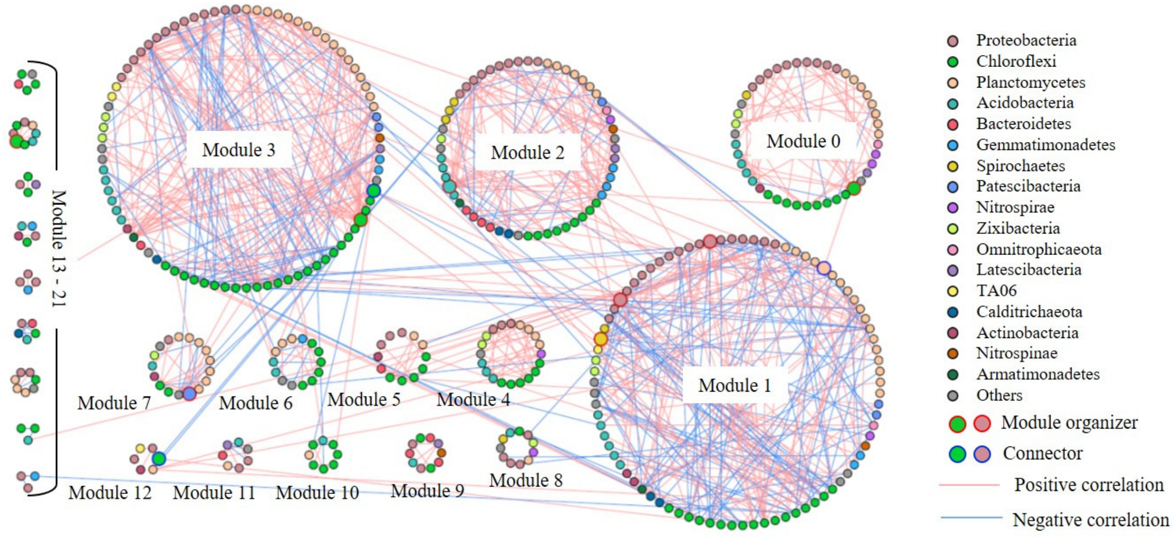

Figure S12. Network of microbial community of BN group.

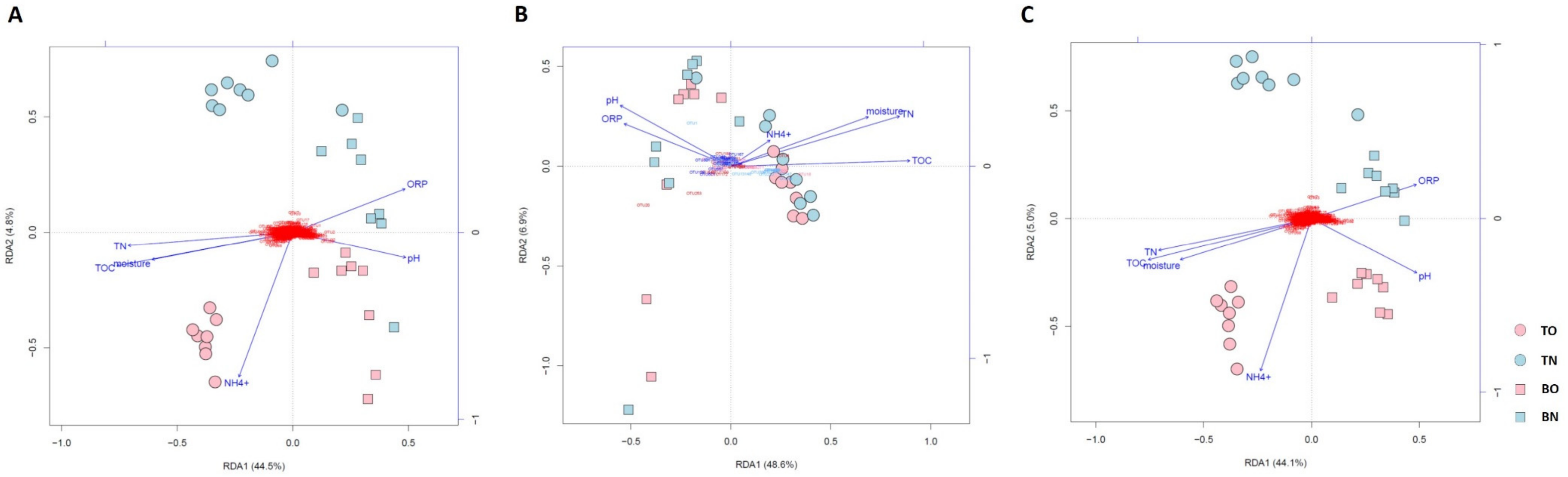

Figure S13. Redundancy analysis based on different sets of microbes. (A) The whole community; (B) key species; (C) all without key species. Blue arrows stand for environmental parameters. In (B), key species from different groups are in different colors (pink: TO; red: BO; light blue: TN; dark blue: BN).

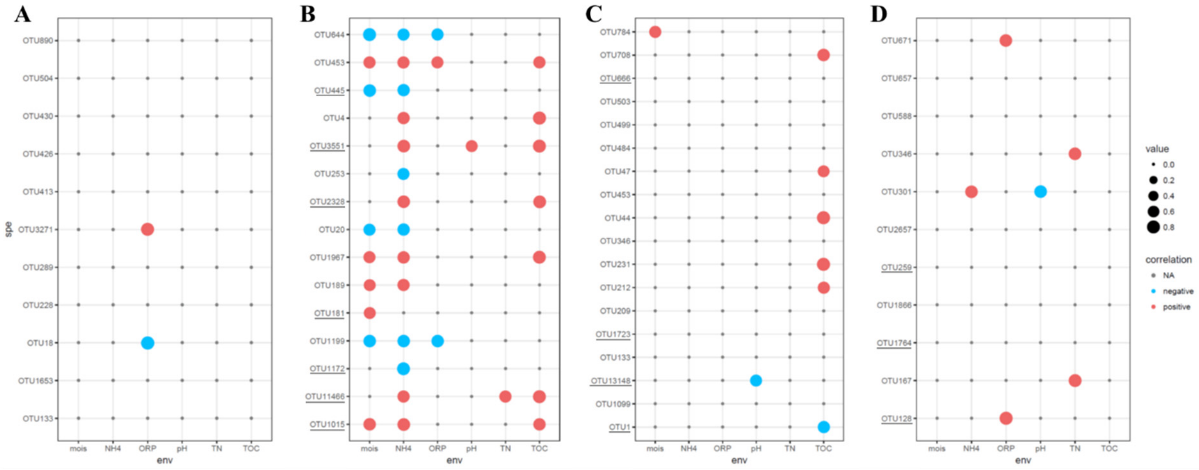

Figure S14. Relativity of keystone species to environmental parameters. (A) TO; (B) BO; (C) TN; (D) BN. Relativity of key OTUs from any given group to environmental parameters. Only significant relativities were depicted. Underlined OTUs were connecters (with high P_i value) and the rest were hubs (with high Z_i value).

Table S6. Taxonomy of the keystone species OTUs.

ID	phylum	class	order	family	genus
OTU3551	Acidobacteria	Aminicenantia	Aminicenantales		
OTU181	Acidobacteria	Thermoanaerobaculia	Thermoanaerobaculales	Thermoanaerobaculaceae	Subgroup_10
OTU2328	Chloroflexi	Dehalococcoidia	GIF3		
OTU1172	Latescibacteria				
OTU1015	Planctomycetes	Phycisphaerae	MSBL9	SG8-4	
OTU445	Proteobacteria	Deltaproteobacteria	Sva0485		
OTU11466	Proteobacteria	Deltaproteobacteria	Syntrophobacterales	Syntrophobacteraceae	
OTU1967	Chloroflexi	Anaerolineae	Anaerolineales	Anaerolineaceae	Pelolinea
OTU4	Chloroflexi	Anaerolineae	Anaerolineales	Anaerolineaceae	
OTU1199	Chloroflexi	Dehalococcoidia	MSBL5		
OTU644	Nitrospirae	Thermodesulfovibrionia			
OTU253	Proteobacteria	Deltaproteobacteria	Desulfarculales	Desulfarculaceae	Desulfatiglans
OTU189	Proteobacteria	Deltaproteobacteria	NB1-j		
OTU20	Proteobacteria	Deltaproteobacteria	Sva0485		
OTU453	Proteobacteria	Deltaproteobacteria			
OTU426	Acidobacteria	Subgroup_22			
OTU504	Acidobacteria	Subgroup_22			
OTU133	Chloroflexi	Anaerolineae	Anaerolineales	Anaerolineaceae	
OTU1653	Chloroflexi	Anaerolineae	Anaerolineales	Anaerolineaceae	
OTU890	Chloroflexi	Anaerolineae	SBR1031		
OTU289	Chloroflexi	Dehalococcoidia	MSBL5		
OTU413	Proteobacteria	Alphaproteobacteria			
OTU228	Proteobacteria	Deltaproteobacteria	Desulfobacterales	Desulfobulbaceae	Desulfobulbus
OTU18	Proteobacteria	Gammaproteobacteria	B2M28		
OTU3271	Bacteroidetes	Bacteroidia	Flavobacteriales	Flavobacteriaceae	Robiginitalea
OTU430	Planctomycetes	Phycisphaerae	MSBL9	SG8-4	
OTU503	Acidobacteria	Subgroup_18			
OTU301	Acidobacteria	Subgroup_22			
OTU484	Acidobacteria	Thermoanaerobaculia	Thermoanaerobaculales	Thermoanaerobaculaceae	Subgroup_10
OTU209	Bacteroidetes	Bacteroidia	Bacteroidales	Bacteroidetes_BD2-2	
OTU499	Bacteroidetes	Bacteroidia	Bacteroidales	Bacteroidetes_BD2-2	
OTU13148	Bacteroidetes	Bacteroidia	Cytophagales	Cyclobacteriaceae	
OTU1866	Chloroflexi	Anaerolineae	ADurb.Bin180		
OTU128	Chloroflexi	Anaerolineae	Anaerolineales	Anaerolineaceae	
OTU167	Chloroflexi	Anaerolineae	Anaerolineales	Anaerolineaceae	
OTU259	Chloroflexi	Anaerolineae	Anaerolineales	Anaerolineaceae	
OTU2657	Chloroflexi	Anaerolineae			
OTU44	Epsilonbacteraeota	Campylobacteria	Campylobacterales	Sulfurovaceae	Sulfurovum
OTU346	Patescibacteria				
OTU1764	Planctomycetes	Planctomycetacia	Pirellulales	Pirellulaceae	
OTU657	Proteobacteria	Deltaproteobacteria	Desulfobacterales	Desulfobacteraceae	SEEP-SRB1
OTU708	Proteobacteria	Deltaproteobacteria	Desulfobacterales	Desulfobacteraceae	
OTU588	Proteobacteria	Deltaproteobacteria	Myxococcales	PS-B29	
OTU666	Proteobacteria	Deltaproteobacteria	NB1-j		
OTU1723	Proteobacteria	Deltaproteobacteria	NKB15		
OTU1099	Proteobacteria	Deltaproteobacteria	Sva0485		
OTU1	Proteobacteria	Deltaproteobacteria	Syntrophobacterales	Syntrophobacteraceae	
OTU212	Proteobacteria	Gammaproteobacteria	Chromatiales	Sedimenticolaceae	Sedimenticola
OTU47	Proteobacteria	Gammaproteobacteria	Chromatiales	Chromatiaceae	
OTU231	Proteobacteria	Gammaproteobacteria	Run-SP154		
OTU671	Spirochaetes	Spirochaetia	Spirochaetales	Spirochaetaceae	
OTU784	Zixibacteria				

Table S7. βNTI of the keystone species of the microbial community.

	TO.2	TO.3	TO.4	TO.5	TO.6	TO.7	TO.8	BO.1	BO.2	BO.3	BO.4	BO.5	BO.6	BO.7	BO.8	TN.1	TN.2	TN.3	TN.4	TN.5	TN.6	TN.7	TN.8	BN.1	BN.2	BN.3	BN.4	BN.5	BN.6	BN.7	BN.8
**TO.1**	-0.17	1.43	1.63	1.28	1.12	2.26	-1.07	-0.68	1.69	0.00	1.02	1.94	0.00	-0.42	2.34	0.32	-0.04	-0.08	0.42	0.39	-0.43	0.52	0.21	1.25	0.08	2.67	-1.16	-1.04	2.18	-0.61	2.46
**TO.2**		1.56	0.16	0.86	0.27	1.13	-0.02	-0.45	1.49	0.60	0.26	0.83	0.71	-0.43	2.09	0.60	0.28	-0.28	-0.07	0.08	-0.69	-0.15	-0.39	1.14	-0.20	2.68	-1.18	-1.15	2.54	-0.63	2.40
**TO.3**			2.46	2.38	2.72	0.27	1.51	0.08	2.85	0.53	2.24	2.31	2.24	0.51	3.10	2.09	1.50	0.24	0.73	-0.22	-0.36	-0.25	-0.50	1.98	-0.02	2.42	-1.26	-1.17	2.63	1.59	3.28
**TO.4**				1.21	1.32	2.16	1.92	2.66	2.54	1.21	0.62	1.22	2.20	1.19	1.99	2.74	0.85	0.42	2.17	0.54	1.64	2.37	0.94	1.14	-0.33	3.58	-0.32	-0.26	3.70	0.40	3.36
**TO.5**					1.19	2.94	0.03	0.85	1.30	1.91	1.59	1.97	2.73	0.58	1.56	3.09	1.97	0.29	2.16	0.66	2.64	3.32	2.98	1.55	-0.30	2.90	0.02	0.19	3.12	0.96	2.60
**TO.6**						2.52	1.23	1.26	2.81	1.13	0.60	1.18	2.43	0.68	1.83	2.57	1.87	0.13	2.25	0.52	2.40	3.41	2.55	1.03	-0.78	3.75	-0.45	-0.34	3.74	0.64	3.53
**TO.7**							0.99	0.98	2.78	2.26	2.19	2.45	2.26	0.32	2.96	2.07	0.64	1.18	1.04	-0.14	0.30	0.26	0.02	1.42	0.28	2.44	-1.18	-1.11	2.75	1.47	3.05
**TO.8**								0.33	-0.17	-1.26	-0.78	-1.01	-1.20	-1.15	0.89	-1.36	0.45	0.09	-0.38	-0.05	-0.69	0.11	0.61	0.77	-1.15	2.03	-0.68	-1.44	0.72	-1.58	-0.41
**BO.1**									1.53	-0.68	-0.43	-0.45	-0.68	-0.74	0.71	-0.59	-0.14	-0.62	0.58	0.09	-0.42	-0.30	-0.49	0.52	0.00	2.51	-0.33	-1.25	1.52	-0.67	0.73
**BO.2**										2.24	2.36	2.10	0.83	1.54	-0.85	0.59	-0.28	-0.37	0.16	0.08	-0.18	1.96	-0.05	0.81	-1.31	1.95	2.59	-0.35	-0.34	0.18	0.21
**BO.3**											0.43	1.94	0.00	-0.42	1.61	0.32	0.28	-0.15	0.48	0.31	0.09	0.46	-0.03	0.74	0.50	0.76	1.72	-0.54	1.51	-0.39	0.53
**BO.4**												-0.44	1.02	1.93	2.37	1.72	0.75	-0.09	0.51	0.85	-0.15	2.13	1.44	1.16	1.42	2.41	1.96	1.00	2.35	1.47	1.76
**BO.5**													1.94	0.95	1.09	1.30	0.93	0.37	0.61	0.69	0.05	2.05	1.12	0.96	0.89	2.22	2.00	1.13	1.84	1.16	1.01
**BO.6**														-0.42	0.00	0.32	0.11	-0.39	0.52	0.37	-0.73	1.60	0.30	0.55	-0.16	2.44	2.08	-0.77	0.94	-0.42	1.63
**BO.7**															1.11	0.24	-0.31	-0.90	0.35	0.36	-0.61	-0.20	-0.83	0.88	-0.49	2.59	0.35	-1.19	1.10	-0.32	1.28
**BO.8**																-0.05	0.62	-0.17	1.35	0.65	-0.02	2.06	1.22	1.31	-0.97	1.84	2.14	-0.94	-0.58	-0.51	-0.38
**TN.1**																	0.02	-0.66	0.12	0.13	-0.73	1.06	-0.45	0.74	0.08	2.51	2.53	0.60	1.74	0.00	2.45
**TN.2**																		0.56	1.16	-0.45	-0.56	1.35	0.64	1.29	-0.77	1.40	-0.84	-0.83	1.21	-0.74	1.92
**TN.3**																			0.61	0.76	0.58	-0.74	0.98	0.42	-0.51	0.36	-0.99	-1.33	0.19	-0.81	0.46
**TN.4**																				0.81	0.37	0.77	-0.09	1.36	-0.08	1.70	-0.40	-0.44	0.81	-0.54	0.95
**TN.5**																					-0.64	0.14	0.09	1.37	-0.08	2.64	-0.11	-0.32	1.41	-0.52	1.23
**TN.6**																						1.52	0.17	1.13	-1.04	0.74	-1.19	-1.14	0.89	-1.07	-0.09
**TN.7**																							-1.02	1.85	0.06	1.66	-0.56	-0.34	2.42	1.14	2.30
**TN.8**																								1.54	-1.06	-0.03	-1.17	-1.04	-0.02	-0.33	1.17
**BN.1**																									0.62	2.18	2.01	0.55	1.08	1.14	0.83
**BN.2**																										1.26	2.04	0.50	-1.03	0.07	-0.87
**BN.3**																											0.44	1.17	2.12	2.18	2.22
**BN.4**																												2.45	2.29	2.20	2.42
**BN.5**																													0.13	-0.41	0.02
**BN.6**																														-0.27	0.20
**BN.7**																															0.21

Table S8. RC$_{bray}$ of the keystone species of the microbial community.

	TO.2	TO.3	TO.4	TO.5	TO.6	TO.7	TO.8	BO.1	BO.2	BO.3	BO.4	BO.5	BO.6	BO.7	BO.8	TN.1	TN.2	TN.3	TN.4	TN.5	TN.6	TN.7	TN.8	BN.1	BN.2	BN.3	BN.4	BN.5	BN.6	BN.7	BN.8
**TO.1**	0.43	0.91	0.8	0.56	0.74	0.91	0.52	0.98	0.94	1	0.97	0.97	1	0.98	0.92	0.97	0.91	0.71	0.86	0.91	0.78	0.34	-0.11	0.9	0.94	0.88	0.87	0.91	0.96	0.96	0.93
**TO.2**		0.83	0.32	0.5	0.59	0.67	0.71	0.9	0.98	1	1	1	1	0.98	0.99	0.99	0.81	0.69	0.81	0.84	0.82	0.7	0.06	0.98	1	0.92	0.81	0.96	0.99	1	1
**TO.3**			0.58	0.76	0.74	0.9	0.67	0.86	0.91	0.99	0.94	0.96	0.97	0.97	0.98	0.9	0.81	0.75	0.81	0.81	0.72	0.28	0.1	0.97	0.83	0.8	0.79	0.8	0.9	0.86	0.89
**TO.4**				0.44	0.46	0.81	0.6	0.94	0.94	1	0.97	0.96	1	0.96	0.95	0.97	0.79	0.65	0.73	0.79	0.75	0.55	0.03	0.92	0.95	0.86	0.79	0.91	0.92	0.95	0.94
**TO.5**					0.66	0.83	-0.33	0.83	0.96	1	0.95	0.96	0.99	0.97	0.95	0.94	0.83	0.7	0.76	0.8	0.69	0.31	-0.4	0.92	0.86	0.8	0.8	0.82	0.92	0.94	0.92
**TO.6**						0.76	0.66	0.94	0.99	1	1	1	1	0.99	1	0.99	0.62	0.57	0.71	0.75	0.74	0.58	0.07	1	0.99	0.92	0.79	0.94	1	1	0.99
**TO.7**							0.88	0.95	0.99	1	1	1	1	0.99	1	1	0.87	0.79	0.86	0.88	0.87	0.75	0.33	0.99	1	0.89	0.85	0.95	0.99	1	1
**TO.8**								0.89	0.94	1	0.94	0.96	0.97	0.97	0.99	0.9	0.81	0.69	0.76	0.83	0.65	0.23	-0.8	0.98	0.85	0.76	0.8	0.82	0.93	0.88	0.89
**BO.1**									0.88	0.97	0.94	0.94	0.96	0.96	1	0.93	0.96	0.92	0.99	0.85	0.87	0.7	0.73	0.97	0.85	0.83	0.84	0.88	0.9	0.87	0.89
**BO.2**										0.88	0.87	0.91	0.1	0.68	1	0.68	1	1	1	0.87	0.88	0.75	0.84	0.96	0.8	0.8	0.92	0.86	0.58	0.77	0.9
**BO.3**											0.94	0.97	1	0.61	1	0.97	1	1	1	0.91	0.91	0.78	0.94	0.99	0.85	0.85	0.99	0.91	0.78	0.87	0.92
**BO.4**												0.92	0.95	0.91	0.84	0.91	1	1	1	0.98	0.99	0.93	0.94	0.66	0.79	0.82	0.98	0.85	0.89	0.82	0.91
**BO.5**													0.95	0.94	0.88	0.95	1	1	1	0.88	0.9	0.75	0.9	0.74	0.78	0.89	0.97	0.89	0.9	0.53	0.88
**BO.6**														0.9	0.97	0.96	1	1	1	0.91	0.91	0.77	0.8	0.87	0.85	0.88	0.94	0.9	0.78	0.5	0.93
**BO.7**															1	0.89	1	1	1	0.87	0.88	0.75	0.9	1	0.81	0.86	0.96	0.86	0.84	0.8	0.9
**BO.8**																1	1	1	1	1	1	1	0.99	-0.17	0.87	0.98	0.98	0.99	1	0.79	0.85
**TN.1**																	1	1	1	0.89	0.91	0.74	0.73	0.97	0.81	0.86	0.83	0.86	0.53	0.78	0.92
**TN.2**																		0.56	0.78	0.81	0.64	0.65	0.14	1	1	0.95	0.82	0.96	1	1	1
**TN.3**																			0.21	0.68	0.73	0.64	0.52	1	1	0.96	0.74	0.93	1	1	1
**TN.4**																				0.77	0.8	0.67	0.65	1	1	1	0.75	1	1	1	1
**TN.5**																					0.75	0.67	0.64	1	0.82	0.81	0.77	0.85	0.85	0.79	0.87
**TN.6**																						0.58	0.49	1	0.83	0.77	0.83	0.84	0.85	0.88	0.86
**TN.7**																							0.47	1	0.66	0.64	0.68	0.73	0.68	0.69	0.7
**TN.8**																								1	0.78	0.63	0.63	0.64	0.79	0.92	0.73
**BN.1**																									0.79	0.94	0.95	0.9	0.97	0.77	0.75
**BN.2**																										0.73	0.87	0.78	0.79	0.41	0.55
**BN.3**																											0.77	-0.33	0.69	0.79	0.82
**BN.4**																												0.78	0.9	0.87	0.87
**BN.5**																													0.79	0.79	0.83
**BN.6**																														0.79	0.87
**BN.7**																															0.56

Table S9. Significant correlation of both all and keystone species to environmental factors in each group.

	Foctors	TO	BO	TN	BN
All species	Moisture	48	222	86	111
	TOC%	50	197	196	68
	TN%	36	64	60	127
	pH	53	62	75	65
	ORP	68	110	33	94
	NH_4^+	50	249	16	83
	SC	305	904	466	548
	SN	1195	1195	1195	1195
	Rate	0.2552	0.7565	0.3900	0.4586
Keystone species only	Moisture		9	1	2
	TOC%		7	6	
	TN%		1		2
	pH		1		1
	ORP	2	3	1	2
	NH_4^+		14		1
	SC	2	35	8	8
	SN	11	15	18	11
	Rate	0.1818	2.3333	0.4444	0.7273

SC: sum of all significant correlations in a given group.

SN: species number in a group.

Rate: calculated from SC divided by SN.

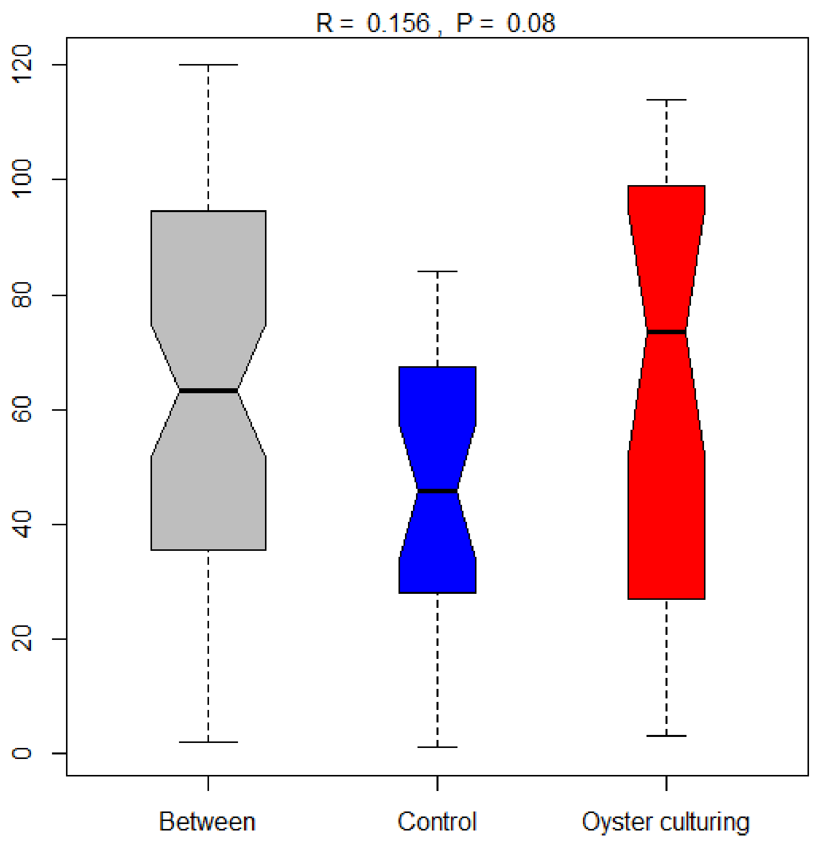

Figure S15. ANOSIM analysis of water parameters. Differences between, gray, and within groups (oyster culturing, red; control, blue). (n=8, $p < 0.05$. Error bar, mean ± s.d.)

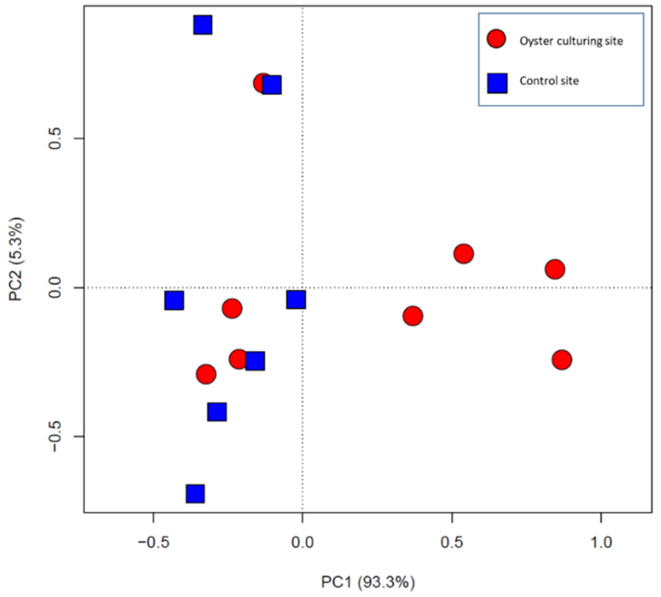

Figure S16. PCA of water parameters. Each point is one sample. Environmental parameters are shown as red arrows. Percentages in parentheses are proportions of difference between parameters represented by the PC axis.

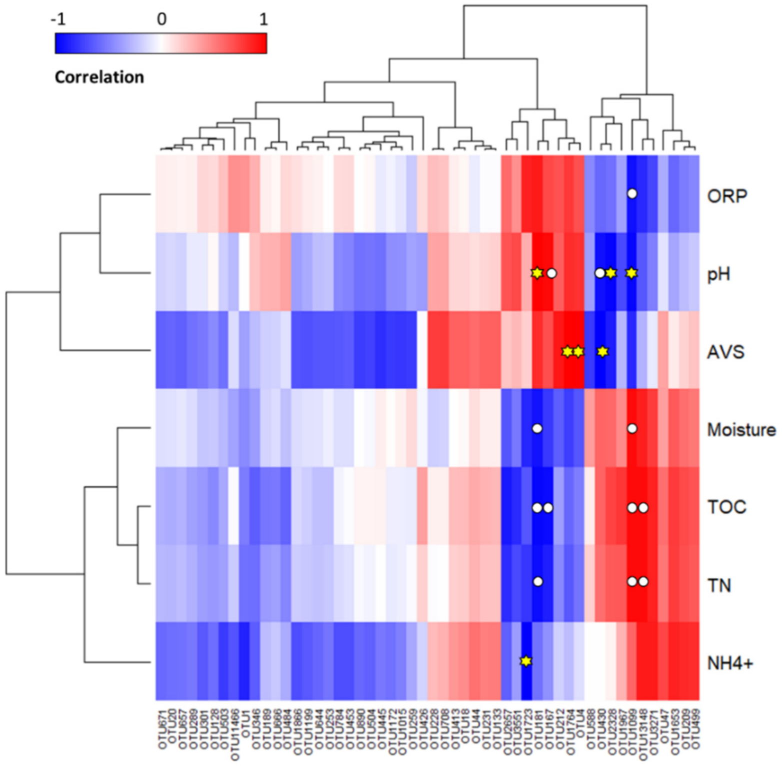

Figure S17. Correlation and significance of keystone species and environmental parameters. Red color means the according OTU and parameter are positively related while blue color indicates negative correlation. Each point is one sample. Significant correlations are marked with yellow stars ($p \leq 0.05$, n=4) and white circles ($p \leq 0.1$, n=4).

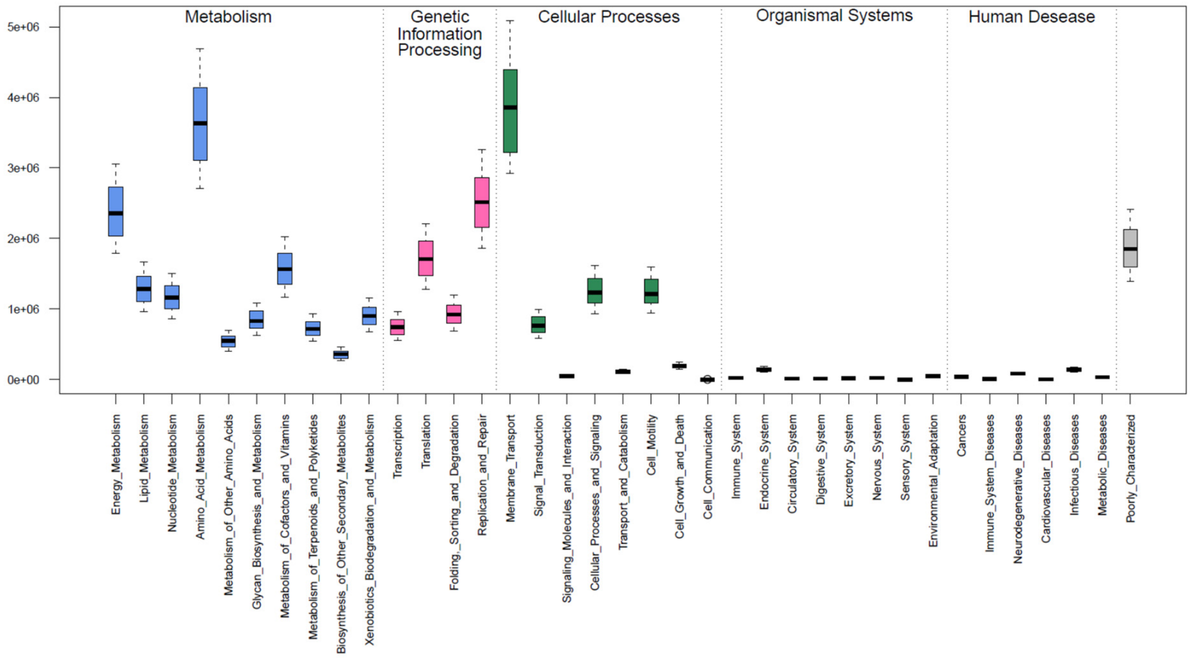

Figure S18. Genome prediction using PICRUSt based on KEGG database. Grey box stands for poorly characterized category.

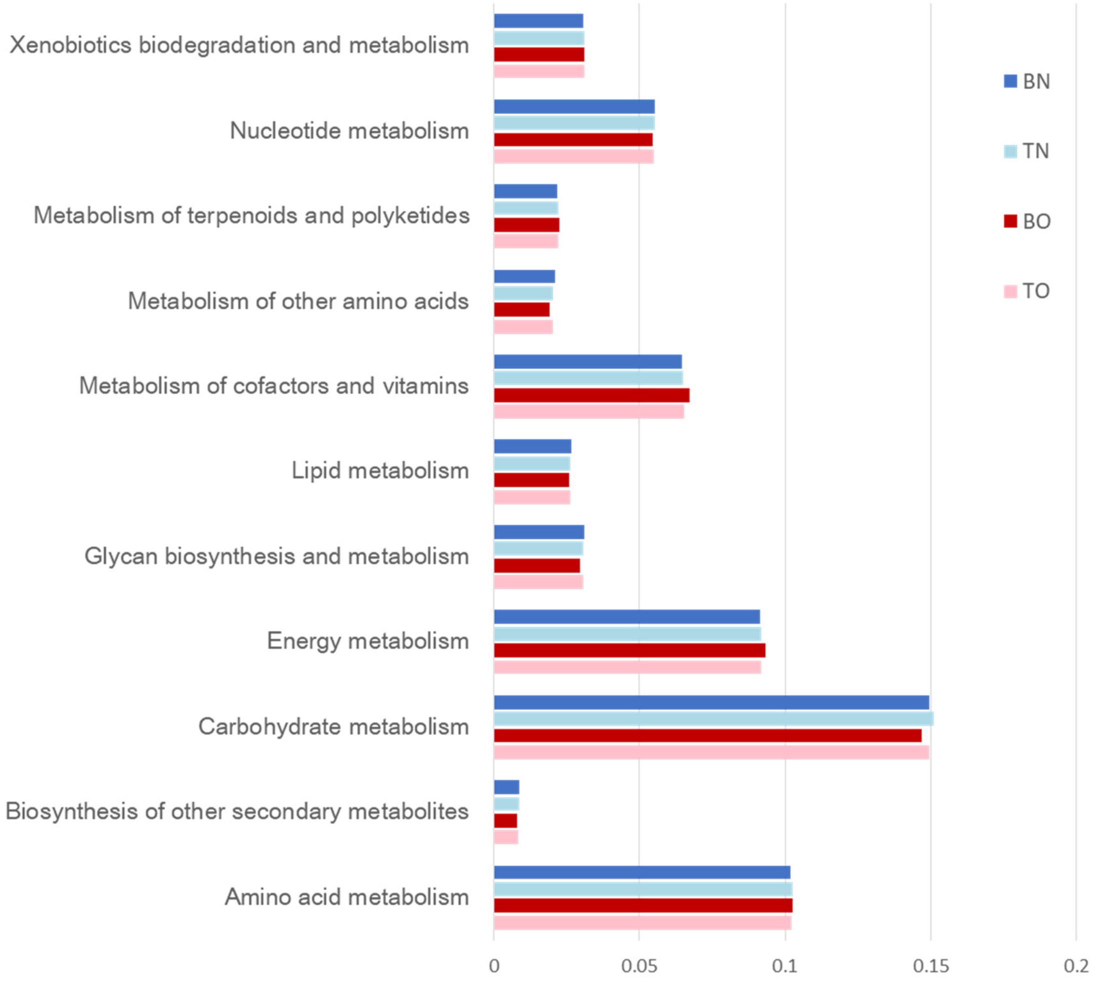

Figure S19. Metabolism contribution prediction of keystone species.
